# Lymph Nodes-On-Chip: Promising Immune Platforms for Pharmacological and Toxicological Applications

**DOI:** 10.3389/fphar.2021.711307

**Published:** 2021-08-16

**Authors:** Aya Shanti, Nicholas Hallfors, Georg A Petroianu, Lourdes Planelles, Cesare Stefanini

**Affiliations:** ^1^ Healthcare Engineering Innovation Center, Biomedical Engineering Department, Khalifa University of Science and Technology, Abu Dhabi, United Arab Emirates; ^2^ College of Medicine and Health Sciences, Khalifa University of Science and Technology, Abu Dhabi, United Arab Emirates

**Keywords:** organs-on-chip, lymph node, immunity, pharmacology, toxicology, lymph node-on-chip

## Abstract

Organs-on-chip are gaining increasing attention as promising platforms for drug screening and testing applications. However, lymph nodes-on-chip options remain limited although the lymph node is one of the main determinants of the immunotoxicity of newly developed pharmacological drugs. In this review, we describe existing biomimetic lymph nodes-on-chip, their design, and their physiological relevance to pharmacology and shed the light on future directions associated with lymph node-on-chip design and implementation in drug discovery and development.

## Introduction

Organs-on-chip have recently emerged as powerful screening tools in the field of pharmacology and toxicology (Maharjan et al., 2020). Such chips enable more accurate evaluation of the safety and efficacy of investigational drugs compared to traditional tissue culture plates and animal models (Low et al., 2020). This is particularly important since the pharmaceutical industry faces high attrition rates for novel drug candidates (Roberts et al., 2014). It is estimated that only 1 out of 10,000 new chemical entities gains FDA approval and transitions it into the market (Nam et al., 2015). Additionally, the overall failure rate in drug development is reported to be over 96%, including a 90% failure rate during clinical development (Hingorani et al., 2019). These numbers, indeed, pinpoint that the current drug development practice suffers from major drawbacks, among which, is the lack of physiologically relevant platforms for drug screening during preclinical evaluation phases (Liu et al., 2017). Organs-on-chip, therefore, provide promising alternatives to conventional preclinical drug screening platforms.

Organs-on-chip are perfused microfluidic chips composed of micro-sized channels wherein cells of a single type or more are allowed to grow and expand in an *in vivo* like microenvironment. They are not intended to construct whole living organs but instead to build the functional units of the organs that are capable of replicating specific aspects of human physiology *in vitro* (Richardson et al., 2020). In particular, they tend to replicate 1) the 3D microarchitecture of the organ, that is, the spatial distribution of its different cellular types; 2) the biochemical microenvironment of the organ including its extracellular matrix as well as its chemokine, growth factor and nutrient gradients, if any; 3) mechanical microenvironment of the organ including mechanical compressions, cyclic strains and shear stresses and 4) particular function of the organ, for example, filtration, respiration or digestion (Shanti et al., 2018). Once an organ-on-chip is established, it can then be used to investigate organ physiology both in health and in disease.

Organs-on-chip can be fabricated using various engineering techniques such as micromolding, microetching, soft lithography and photo-polymerization (Shanti et al., 2018). These techniques have enabled the development of extremely intricate structures that closely resemble those *in vivo* and these include the villi of the small intestine and the alveoli of the lungs (Shim et al., 2017; Stucki et al., 2018).

Over the past decade, many research groups have developed different organs-on-chip and demonstrated their ability to be used in pharmacological, toxicological and drug testing applications. For instance, Hassell and others developed a lung-on-chip platform that replicates lung’s microenvironment-specific cancer growth and lung’s response to tyrosine kinase inhibitor therapy (Hassell et al., 2017). In addition, Vernetti et al. developed a liver-on-chip platform containing primary human hepatocytes along with human endothelial cells, immune cells and stellate cells and assessed the toxicity of various molecules (troglitazone, nimesulide, caffeine, trovafloxacin, levofloxacin, LPS and methotrexate) on the developed liver model (Vernetti et al., 2016). Moreover, Jalili-Firoozinezhad et al. developed a gut-on-chip platform lined by human intestinal epithelial cells and vascular endothelial cells, utilized it to model radiation injury and then deployed it to assess the efficacy of a radiation countermeasure drug (prolylhydroxylase inhibitor) (Jalili-Firoozinezhad et al., 2018). Furthermore, Qian et al. developed a heart-on-chip platform capable of recording cardiac tissue adhesion, electrophysiology, and contractility and used it to study the effect of norepinephrine on the heart (Qian et al., 2017). Still, many other organ-on-chip platforms remain and have been utilized to investigate different processes such as mechanism of action of drugs, pharmacokinetics and pharmacodynamics, efficacy, toxicity and dose response (Richardson et al., 2020).

Despite the development of many organs-on-chip and their utilization in pharmacological and toxicological contexts, only recently has the need for developing a lymph node-on-chip been recognized. This comes after acknowledging the fact that the immune outcome from the lymph node is a key determinant of not only the response to viruses, bacteria, and other foreign particles but also of the immunotoxicity of drugs, i.e. toxicity of the drugs to the immune system (Galarza et al., 2020). Lymph nodes-on-chip can facilitate investigations into the mechanisms of interaction between immune cells and drug candidates thus, unveiling significant knowledge to cut down the high cost of drug development as well as to reduce the high attrition rate.

In this review, we describe the human native lymph node, summarize existing biomimetic lymph nodes-on-chip, their design, and their physiological relevance to pharmacology and shed the light on future directions associated with lymph node-on-chip design and implementation in drug discovery and development.

## Human Native Lymph Node

Lymph nodes are vital organs of the immune system where efficient and protective immune responses are generated and maintained (Gasteiger et al., 2016; Novkovic et al., 2020). Lymph nodes collect and filter the fluid that drains from peripheral tissues, known as the lymphatic fluid, before it is eventually returned into blood circulation (Fullerton and Gilroy, 2016). This process is essential to achieve an efficient adaptive immune response and to protect the body against foreign bodies such as bacteria and viruses.

**FIGURE 1:**
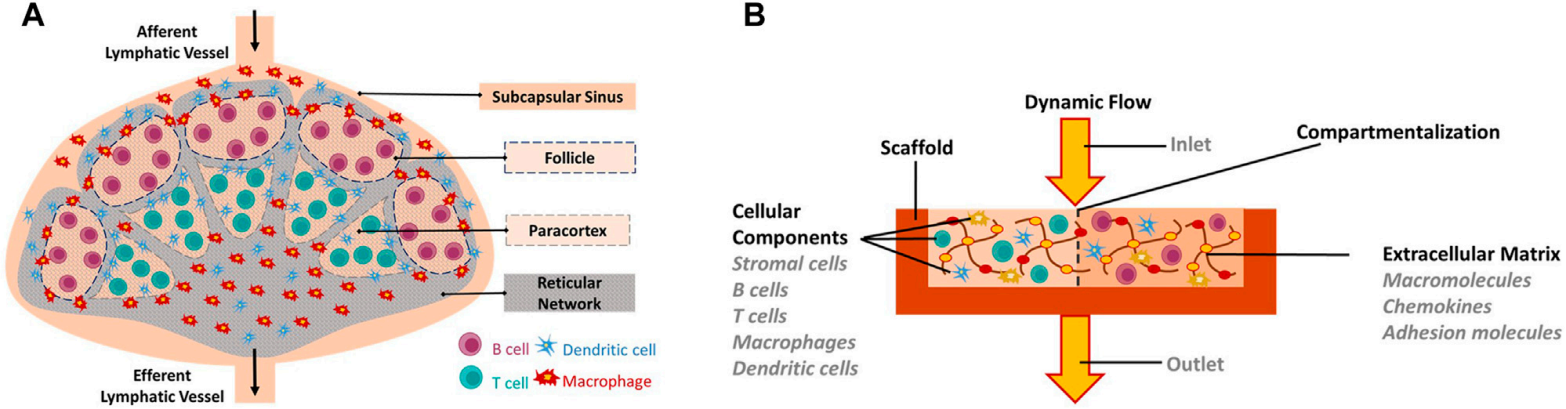
*In vitro* engineering of human Lymph node-on-a-Chip. **(A)** Schematic of the human native lymph node. **(B)** Requirements for *in vitro* biomimetic lymph node-on-chip. Requirements include: Biomimetic scaffold on which cells can be seeded or grown, cellular components (immune cells and stromal cells), appropriate extracellular matrix components, dynamic flow conditions, key features of the native lymph node including compartmentalization.

Lymph nodes provide unique structural microenvironments to support efficient gathering of immunogenic material from peripheral tissues (Figure 1) (Sainte-Marie, 2010; Schudel et al., 2019; Shanti et al., 2020). They are compartmentalized into distinct cellular micro-domains populated by a single type of lymphocytes, either B lymphocytes or T lymphocytes (Crivellato et al., 2004; Cupedo et al., 2012). B lymphocytes populate an area known as the primary follicle within the outer cortex of the lymph node whereas T lymphocytes populate an area known as the paracortex. When B lymphocytes residing in the follicles are activated by an antigen, they initiate the formation of germinal centers, which are dynamic microenvironments where B lymphocytes proliferate, differentiate and generate high affinity antibodies necessary for an effective immune response (Domeier et al., 2017).

Not all molecules entering the lymph node have free access to the distinct cellular compartments (follicle and paracortex). Instead, when the lymphatic fluid enters the lymph node through the afferent lymphatic vessel, it is directed to an area known as the subcapsular sinus (SCS) rather than being directly directed to the cellular regions (von Andrian and Mempel, 2003). SCS contains macrophages that sample the lymphatic fluid and remove any microorganisms and debris found in it. In addition, macrophages process antigenic material found in the lymphatic fluid and present it to B and T lymphocytes residing in their specific compartments (von Andrian and Mempel, 2003). As the lymphatic fluid flows through the SCS, a small fraction of it is diverted laterally into a conduit system formed by reticular collagen fibrils that penetrate the B follicle and reach deep into the paracortex (Jafarnejad et al., 2015; Shanti et al., 2020). The conduit system delivers antigens into the follicle and paracortex (Gretz et al., 2000; Liao and von der Weid, 2015; Hughes et al., 2016). In addition, it constitutes a scaffold on which immune cells can migrate and acts as a filtration system (Katakai, 2004). It has been shown that the conduit system only allows small molecules, whose diameter is between 7.2 and 10.7 nm to pass through and penetrate into the follicle or paracortex (Gretz et al., 2000). In their specific compartments, T and B lymphocytes scan the incoming molecules and/or cells for their cognate antigens. If such antigens are not found, cells receive homeostatic survival signals (Cupedo et al., 2012). Conversely, if their cognate antigens are found, cells are activated and induced to proliferate and differentiate: T-cells into cytotoxic (CD8^+^) effector T-cells, helper (CD4^+^) effector T-cells or memory cells and B-cells into plasma cells or memory cells (Shanti et al., 2018). Most activated cells then leave the lymph node and head towards the site of infection to eliminate the foreign antigens (Pape et al., 2007). Remaining lymphatic fluid and soluble molecules, which are not directed into the conduit system, are transported through a number of small trabecular sinuses and medullary sinuses, after which they leave the LN through efferent lymphatic vessels.

The main extracellular component of the lymph nodes is collagen Type I (Kaldjian et al., 2001; Wiig et al., 2010). Furthermore, the organization of the lymph node into distinct cellular regions is controlled by various chemokines and adhesion molecules such as Chemokine (C-C motif) ligand 19 (CCL19), Chemokine (C-C motif) ligand 21 (CCL21), C-X-C Motif Chemokine Ligand 12 (CXCL12) and the selectively chemotactic ligand for B cells CXCL13 (B-cell-attracting chemokine 1) expressed on stromal cells (Cyster, 1999; Katakai, 2004; Lian and Luster, 2015). Besides the secretion of chemokines and adhesion molecules, stromal cells provide functional support to the lymph node (Katakai, 2004). In particular, fibroblastic reticular cells produce various extracellular matrix components and interweave them to make reticular fibers, which in turn, provide mechanical strength to the lymph node, allow migration of immune cells from one area to another and form the conduit system necessary for the filtration of the lymphatic fluid (Gretz et al., 1996, 1997; Mcknight and Gordon, 1998). Dynamic migration processes in the lymph node enable cellular communication between antigen presenting cells (APC), B cells and T cells, which is critical in the generation of the adaptive immune response (Allen et al., 2007).

### TECHNOLOGIES FOR GENERATING LYMPH NODES-ON-CHIP

The design and development of an *in vitro* lymph node model has several aspects to be considered and these include: the development of a biomimetic scaffold on which immune cells can be seeded or grown, the generation of a microenvironment with appropriate extracellular matrix components, the production of dynamic flow conditions and the replication of key functional features of the native lymph node (Figure 1). Therefore, researchers have employed various technologies for reproducing the lymph node *in-vitro*. To achieve a biomimetic scaffold on which cells can be seeded or grown, many relied on soft lithography of SU-8 to create molded polydimethylsiloxane (PDMS) microfluidic devices (Nandagopal et al., 2011; Mitra et al., 2013; Moura Rosa et al., 2016; Parlato et al., 2017; Ross and Pompano, 2018; Shim et al., 2019). These devices can be single a layer bonded to glass, or multiple layers, bonded together to form a central channel. In addition to silicone, some designs utilized other materials such as gelatin, polysulfone, or agarose to form fluidic pathways (Giese et al., 2006, Giese et al., 2010; Haessler et al., 2009). Microfluidics, in turn, enabled the execution of tasks not possible in larger-scale environments. It allowed the establishment of chemical gradients for chemotaxis studies, enabled the selective capture of small populations of cells for time-lapse microscopy and allowed the measurement of tissue diffusion mechanics (Haessler et al., 2009; Zaretsky et al., 2012; Mitra et al., 2013; Moura Rosa et al., 2016; Ross and Pompano, 2018). Still, the ability to select semi-permeable channel material allowed for broader diffusion and a chemical gradient establishment in the surrounding medium (Haessler et al., 2009).

To achieve a microenvironment that mimics the extracellular matrix of the lymph node, three dimensional hydrogels have been used. These create more biologically relevant conditions *in-vitro*, with the choice of gel material having direct chemical and functional outcomes on cell behavior. The ideal biomaterials should have similar stiffness than the target tissue, degradability, and equivalent ability to support the binding of soluble factors, cellular adhesion, cellular communication, and cellular movement. Extracellular matrix is commonly replicated using hydrogels based on poly-ethylene glycol (PEG), agarose, or collagen I, the last being the main component of the extracellular matrix in the naïve lymph node. Depending on the selection, the hydrogel can be improved by adding other molecules. For example, PEG has been functionalized with maleimide (maleic acid and imide) and incorporated with collagen peptides to improve its binding and adhesion properties (Graney et al., 2020). Fibronectin has been also widely used to recreate the extracellular matrix due to its ability to bind multiple proteins such as proteoglycans, growth factors, integrins and chemokines (Pelletier et al., 2000; Woods et al., 2000; Martino and Hubbell, 2010; Rana et al., 2015).

To achieve dynamic conditions, lymph nodes on-chip require active fluidic perfusion. Thus, many researchers utilized peristaltic pumps to flow cell medium and suspended biological material into their developed models (Giese et al., 2006, 2010; Moura Rosa et al., 2016; Sardi et al., 2016; Shim et al., 2019). Active pumping often induces cellular cross-talk by the transport of chemokines or cell markers (Parlato et al., 2017; Polini et al., 2019; Shim et al., 2019).

Another important aspect in the design of a biomimetic lymph node is its ability to support the co-culture and spatial distribution of the different cell types that populate this organ (mainly immune T and B cells, DCs, and stromal cells) in a similar way to how they are organized in the human model. This represents a big challenge that, if achieved, considerably increases the complexity of the design, the experimental conditions and the data analysis. Most of the studies, so far, have used only one or two immune cell types in their devices. The source of the cellular components is also an important point to consider. Some devices are validated using immortalized human cell lines as they are easy to obtain, manipulate and expand, are cost effective and allow experimental reproducibility (Moura Rosa et al., 2016; Birmingham et al., 2020; Shanti et al., 2020; Hallfors et al., 2021). Cell lines are well established models that can be easily modified to target and study molecules of interest. The use of cell lines to mimic *in vitro* what is occurring *in vivo* in the lymph node has nevertheless clear limitations as immortalized cells fail to fully reproduce morphological and functional characteristics of primary cells. For example, cell lines can’t be used to fully replicate humoral immunity in which naïve B cells need to proliferate, differentiate and become antibody secreting cells. These limitations can be circumvented by the use of primary immune cells commonly purified from human blood and also tissue slices from human lymph nodes (Lin and Butcher, 2006; Higbee et al., 2009; Giese et al., 2010; Dauner et al., 2017; Radke et al., 2017; Goyal et al., 2019; Kraus et al., 2019). These attractive models, which, in fact, are still under development, attempt to be closer to the physiological behavior of the human lymph node. However, they are also challenging in several aspects such as limitation in the amount of material, short lifespan, specific requirements for cell culture, and donor variability. Special attention should be given to human leukocyte antigen (HLA) -mismatch when using co-cultures of cells from different donors, as it could lead to cell activation and misinterpretation of the results.

We will describe next the most comprehensive Lymph node on chip platforms found in the literature and the technology behind.

### Bioreactor Human Artificial Lymph Node

An attempt to model the human lymph node is a commercial perfusion bioreactor system named the Human Artificial Lymph Node reactor (HuALN^®^, ProBioGen AG, Berlin, Germany) or HIRIS^TM^III bioreactor (Giese et al., 2006)*.* It is made of polysulfone (PS), has two culture compartments separated by a double layer of gas-permeable membranes and, two fluidic systems, one for culture medium and one for suspended cells. The HuALN^®^ enables long term culture of cells embedded in agarose matrix and the establishment of well-defined gradients. Authors showed this model is beneficial to study immune reactions and drug responses because they were able to detect micro-organoid structures (by histology and *in situ* imaging), to analyze genetic profiles and, to quantify cytokine and immunoglobulin secretion in the collected cell culture medium (Giese et al., 2010; Radke et al., 2017; Kraus et al., 2019)This model has been used to test glycoprotein vaccines and to study the immunogenicity of protein aggregates of two antibodies, bevacizumab and adalimumab, exposed at different stress conditions (Radke et al., 2017; Kraus et al., 2019).

### Multicompartment Lymph Node-on-a-Chip Model

Recently, our research group at the Healthcare Engineering Innovation Center in Khalifa University, UAE has also developed a novel microfluidic platform replicating multiple key features of the lymph node aimed at facilitating biological investigations of immune cellular kinetics, cell-cell interactions, cell-drug interactions and sampling (Shanti et al., 2020; Hallfors et al., 2021). The developed lymph node-on-chip recreates 1) the spatial microenvironment of the native lymph node, 2) the compartmentalization of immune cells within distinct regions 3) the extracellular microenvironment of the native lymph node 4) the fluid flow within the native lymph node and 5) several functional features of the native lymph node. It is a multi-compartmentalized bioreactor consisting of an elliptical body with multiple compartments resembling the different cellular regions of the native lymph node, an inlet aperture resembling the afferent lymphatic vessel and an outlet aperture resembling the efferent lymphatic vessel. The cellular regions are filled with 3D hydrogels encapsulating immune cells. These hydrogels are collagen based and have morphology, porosity, stiffness, and permeability comparable to that of the native human lymph node and sustain cellular viability for duration sufficient for immunotoxicity studies. To demonstrate its feasibility for pharmacological and toxicological applications, we utilized the lymph node-on-chip to assess the effect of a pharmaceutical drug, namely hydroxychloroquine, on the motility of immune cells and their production of reactive oxygen species. We showed that hydroxychloroquine reduced T cell velocity, promoted persistent rotational motion and increased levels of reactive oxygen species. These results highlight the potential of our lymph-node-on-a-chip to be used in pharmacological applications. They also demonstrate that the lymph node-on-a-chip is an attractive alternative to further dissect and understand the mechanisms of action of drugs delivered to cells. The LN-on-chip is intended for identifying comparative behaviour of immune cells in response to drugs and for recognizing patterns of action.

### Modular Immune *In vitro* Construct

Another promising immune platform, Modular Immune *In vitro* Construct (MIMIC), was developed by Warren et al. for evaluating the immune response to vaccines (Warren et al., 2005, 2007). The platform consists of a peripheral tissue equivalent mimicking the skin from which the vaccine is introduced, a lymphoid tissue equivalent mimicking the lymph node where interactions between immune cells and vaccine components take place and a functional assay module that allows the assessment of the output of the immune response. Researchers utilized MIMIC platform for evaluating immune response to several vaccines including seasonal trivalent influenza vaccine and Tetanus vaccine (Byers et al., 2009; Higbee et al., 2009; Drake et al., 2012; Dauner et al., 2017). In their study with the influenza vaccine, Dauner et al. used blood derived immune cells from young and relatively old human donors and demonstrated the ability of MIMIC platform to recapitulate differential age-associated response to the vaccine. In particular, they showed that immune cells from elderly population exhibited reduced antibody IgG production and reduced CD154 + IFNc + IL-2+TNFa + CD4^+^ T activation upon stimulation with the vaccine compared to young population. Authors concluded that MIMIC platform is an attractive tool to reduce the cost and developmental time of new vaccines and therapeutics.

## Selective features recreated by Lymph Node on chip models

**FIGURE 2:**
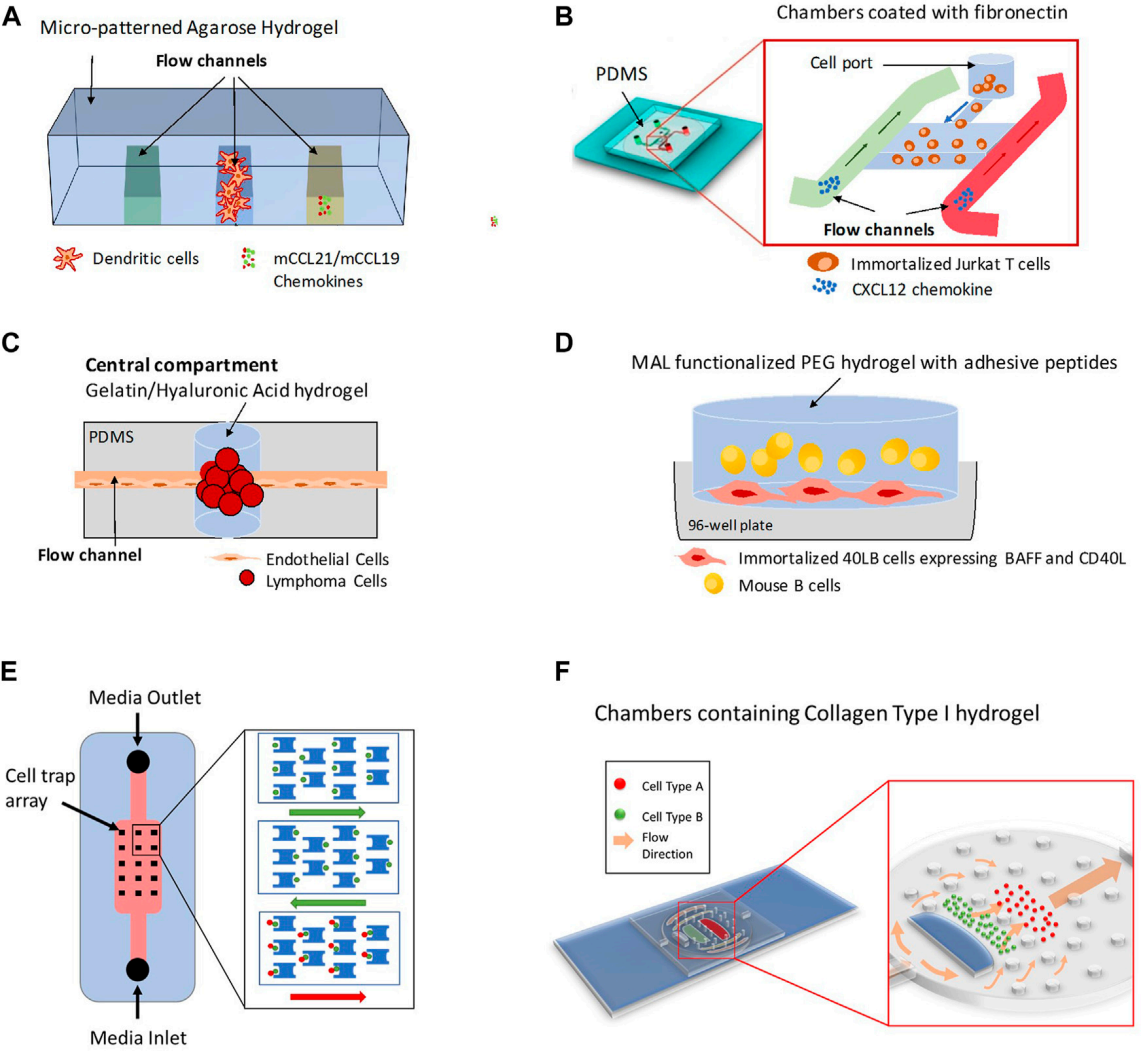
Different 3D Lymph Node-on-chip Models. **(A)** Model for 3D chemotaxis studies by (Haessler et al., 2009). It consists of three channels that produce a chemotactic gradient across the central channel, which is populated by Dendritic cells (DCs). **(B)** Model for flow-free chemokine gradient establishment and T cell migration analysis by (Sonmez et al., 2020). It consists of two flow channels and one microchamber. **(C)** Lymphoma-on-chip model for recreating the *in-vivo* like interactions between immune cells and tumors by (Mannino et al., 2017). The chip consists of a Polydimethylsiloxane (PDMS) structure that houses a hydrogel tumor model and that is traversed by an endothelialized channel. **(D)** Model for replicating germinal center formation, B cell differentiation and class switching recombination *ex-vivo* by (Béguelin et al., 2017; Purwada et al., 2019; Graney et al., 2020). It consists of Maleimide (MAL) functionalized poly (ethylene glycol) (PEG) immune tissue model with suspended B-cells and adherent 40LB cells in a microwell plate. **(E)** Model for quantitative profiling and imaging of lymphocyte interactions by (Dura et al., 2015). It consists of weir-based PDMS cellular traps. **(F)** Model for 3D co-culture of multiple cell types and for investigations of immune cell-cell interactions and immune-drug interactions by (Shanti et al., 2020; Hallfors et al., 2021). It consists of an elliptical body with multiple compartments formed by PDMS micropillars resembling the different cellular regions of the native lymph node, an inlet aperture resembling the afferent lymphatic vessel and an outlet aperture resembling the efferent lymphatic vessel.

Up to the current time, lymph node on-chip models have only recapitulated selective features of the human lymph node. In the following sections, we describe attempts to recreate aspects of the lymph nodes on-chip and shed the light on future directions associated with lymph node-on-chip design and implementation in drug discovery and development. There are other organ-on-chip models that are immune related and that hold promising potential for investigations related to the immune system (Stachowiak and Irvine, 2008; Jones et al., 2012; Dura et al., 2015; Sun et al., 2019; Fathi et al., 2020; Bachmann et al., 2021) but we will focus on models that mimic the lymph node physiology. Table 1 summarizes existing attempts to develop *in vitro* lymph nodes-on-chip. Figure 2 shows some lymph node on-chip models.

**TABLE 1:** Summary of existing attempts to develop *in vitro* lymph nodes on-chip.

Replicated feature of lymph node	Lymph node Scaffolding material	ECM material	Microfluidics	Cellular component	Application	References
Chemotaxis and Chemokine Diffusion	Agarose	-Collagen hydrogel	Yes, active pumping	Mouse bone-marrow derived dendritic cells	Dendritic cell chemotaxis	Haessler et al. (2009), Haessler et al. (2011)
		-Matrigel			Recreation of chemokine gradients (CCL21 and CCL19)	
		-Heparan Sulfate Proteoglycans				
	Photolithography-patterned PDMS	No hydrogel	Yes, active pumping	Mouse bone-marrow derived dendritic cells	Dendritic cell chemotaxis. Recreation of single and competing chemokine gradients (CCL21, CCL19 CXCL12)	Ricart et al. (2011)
		Fibronectin coating of surface				
	Photolithography-patterned PDMS	No hydrogel	Yes, active pumping	Human T cells from blood	-Blood derived T cells migration towards controlled gradients of CCL19 and CXCL12	Lin and Butcher, (2006)
		Fibronectin coating of surface				
	Photolithography-patterned PDMS	No hydrogel	Yes, active pumping	Mouse lymph node slices	Effective diffusion of cytokines in live mouse lymph node slices (TNFα, IL2 and IFNγ)	Ross and Pompano, (2018)
Subcapsular Sinus Dynamics	Adhesive microchannel affixed to a PDMS block previously cured in a polystyrene tissue culture plate	No hydrogel	Yes, active pumping	Thp1 human monocytic cell line	Effect of subcapsular sinus biophysical (flow and structure) and biochemical (adhesion molecule expression) remodeling on cellular adhesion	Birmingham et al. (2020)
		Portion of channel was functionalized by Fc specific anti-IgG		LS174T human colon cancer cell line		
		Plates were blocked with BSA		PANC-1 human pancreatic cell line		
Immune cellular interactions	Photolithography-patterned PDMS	No hydrogel	Yes, active pumping	Mouse primary T and B cells	Assay molecular events during lymphocyte activation	Dura et al. (2015)
		Surface blocking using bovine serum albumin or pluronic F127			Interaction between OT-I CD8 T cells and SIINFEKL-loaded MHCII-eGFP B cells	
					Activation dynamics of CD8 OT-1 T cells and TRP1 transnuclear T cells	
	Photolithography-patterned PDMS	Collagen and fibronectin	Yes, active pumping	-MutuDC: Mouse dendritic cell line	Dynamic interaction of flowing lymphocytes with adherent dendritic cells	Moura Rosa et al. (2016)
				-MF2.2D, OVAII: Mouse CD4 T cell line	Effects of low and high shear stress variations on adhesion	
				-RF33.70/OVAI: Mouse CD8 T cell line		
Germinal center formation and antibody production	Not applicable	-Gelatin activated with silicate nanoparticles	No	-Mouse primary B cells	Immuno-engineered organoids to mimic germinal center formation, class switching and antibody production	Purwada et al. (2015), Purwada et al. (2017); Purwada et al. (2019); Béguelin et al., (2017); Purwada and Singh (2017); Graney et al. (2020)
		-Functionalized poly-ethylene glycol (PEG) that incorporates adhesive peptides		-Fibroblastic cell line expressing CD40L and BAFF molecules		
	Photolithography-patterned PDMS	- Gel composed of Matrigel and collagen I	Yes, active pumping	- Human T and B cells purified from blood	Mimic germinal center formation, class switching and antibody production	Goyal et al. (2019)
Lymph Node Malignancies	Photolithography-patterned PDMS	Thiol-modified Hyaluronic acid, gelatin and PEG	Yes, active pumping	-MLMVECs mouse lung microvascular endothelial cell line	Model that replicates a blood vessel within a tumor and allows the study of complex interactions between immune cells and endothelial cells in the lymphoma microenvironment	Mannino et al. (2017)
				-A20 mouse B-cell lymphoma cell line. Cells used from explanted A20 tumors in mice		
				-Mouse primary CD3^+^ T cells and CD11b + myeloid cells from spleen		
	Photolithography-patterned PDMS	No hydrogel	Yes, active pumping	-Mouse lymph node slices	Model that replicates communication between tumor and lymph node	Shim et al. (2019)
				-Murine 4T1 breast tumors slices	Analysis of protein release and capture between two organs	
Chemical Stimulation	Photolithography-patterned PDMS	Agarose	Yes, active pumping	Mouse lymph node slices	Recreate stimulus-response behavior of the lymph node	Ross et al. (2017)
Response to Drugs and vaccines	Photolithography-patterned PDMS	Collagen	Yes, active pumping	-Jurkat human T cell line	Model that replicates lymph node architecture, ECM components and flow	Shanti et al. (2020); Hallfors et al. (2021)
				-Raji human B cell line	Analysis of cell interactions and drug effects on cell dynamics	
				-Thp1 human monocytic cell line		
	-Polysulfone	Agarose	Yes active pumping (medium and gas)	-Human T and B cells purified from blood	HIRIS^TM^III bioreactor that mimics long term interactions (14 days) between suspended lymphocytes and adherent dendritic cells	Giese et al. (2006), Giese et al. (2010); Sardi et al. (2016); Radke et al. (2017); Kraus et al. (2019)
	-Polycarbonate base plate			-Human dendritic cells derived from blood monocytes	Used to test glycoprotein vaccines and the immunogenicity of protein aggregates	
				-Mesenchymal stromal cells (MSCs)		
	Transwell	-	No	- Human T and B cells purified from blood	Immune response to vaccines including seasonal trivalent influenza vaccine and Tetanus vaccine	Warren et al. (2005); Warren et al. (2007); Higbee et al. (2009); Drake et al. (2012); Dauner et al. (2017)

### Chemotaxis and Chemokine Diffusion

Initial studies using 3D microfluidic platforms that partially replicated the human lymph node were focused on characterizing cellular migration. Chemokines and chemokine receptors are key molecules expressed by multiple cell types in the lymph node such as T lymphocytes, B lymphocytes, and DCs. These molecules not only support cellular organization but also guide immune cells during the immune response to allow them to move between the outer zone where antigens are acquired and the areas where B and T cells are activated.

In 2009, Haessler et al. described an agarose-based microfluidic platform with a gradient buffer for 3D chemotaxis studies (Haessler et al., 2009). This platform had a central cell culture compartment and two side flow channels. DCs were embedded in the central region in a collagen-matrigel-proteoglycans mixture and chemokines and/or media were flowed along the side channels of the device to recreate, in a controlled manner, well defined chemokine gradients. Using this device, authors found that the bone marrow derived murine DCs preferentially migrate towards Chemokine (C-C motif) ligand 21 (CCL21) versus Chemokine (C–C motif) ligand 19 (CCL19) when they are exposed to equivalent and opposite gradients (Haessler et al., 2009). CCL21 and CCL19 are the two endogenous ligands for the chemokine receptor CCR7.

The integration of different chemotactic signals by DCs was also addressed by Ricart el al. through another microfluidic device that similarly allowed the recreation of single and competing chemokine gradients (Ricart et al., 2011). It also enabled the analysis of the effects of actin and myosin inhibitors, as well as pertussis toxin on DC chemotactic migration. This device, described by the authors previously to evaluate neutrophil migration, used fibronectin to favor cell adhesion (Jannat et al., 2010). Authors found that CCL19 signal was more potent than CCL21 or C-X-C motif chemokine 12 (CXCL12) (ligand for receptors CXCR4 and CXCR7, later renamed ACKR3 implying it belongs to the atypical chemokine receptors).

Unlike Haessler et al. and Ricart et al. who studied migration of DCs in microfluidic platforms, Lin et al. were among the first to study blood derived T cells migration towards controlled gradients of CCL19 and CXCL12 using a microfluidic device coated with fibronectin (Lin and Butcher, 2006). Similarly, but more recently, Sonmez et al. developed another PDMS-based microfluidic system able to establish flow-free chemokine gradient patterns and allow T cell migration analysis by time-lapse imaging (Sonmez et al., 2020). Their device had two flow channels and one microchamber for cell culture with the chambers coated with fibronectin to mimic extracellular matrix. Using this system, they generated flow-free concentration gradient by the diffusion of the chemotactic response of Jurkat T cells to CXCL12.

In a different setting, Ross et al. reported a three-layer device to quantify the effective diffusion of cytokines (TNFα, IL2 and IFNγ) in live mouse lymph node slices placed in the cell chamber (Ross and Pompano, 2018). Diffusion was monitored through imaging by time lapse experiments. This model might be applied to study drug delivery and diffusion through the lymph node extracellular matrix.

### Subcapsular Sinus Dynamics

In a different attempt, Birmingham et al. developed a novel microfluidic device to investigate the effect of subcapsular sinus remodeling (seen in inflammation) both biophysically (flow and structure) and biochemically (adhesion molecule expression) on human monocytic and metastatic cellular adhesion (Birmingham et al., 2020). The microfluidic device consists of a divergent adhesive microchannel cut from a 125 mm thick double sided adhesive tape and affixed to a PDMS block previously cured in a polystyrene tissue culture plate. The cells incorporated in the model include THP-1 human monocytic cell line as well as LS174T human colon cancer cell line and PANC-1 pancreatic cell line. The authors showed that decreased wall shear stress usually seen in flow regimes associated with inflamed lymph nodes influences the trajectories and extent of E-selectin mediated cancer cell adhesion. Interestingly, they showed that adhesion of LS174T cells to E-selectin is enhanced by co-perfusion with THP-1 cells. Furthermore, authors demonstrated that adhesion molecules, intercellular adhesion molecule (ICAM) and vascular cell adhesion molecule (VCAM) may enable cancer cell homing to the lymph nodes through the modulation of the extent and dynamics of cellular adhesion. Taken together, these results emphasize that lymph node remodeling plays a significant role in cancer invasion and lymphatic metastasis.

### Immune Cellular Interactions

Microfluidic devices have been also used to understand the cellular interactions and the physiological environment in the lymph node. Dura et al. developed a unique microfluidic device that trapped cells at a pair level and at a single cell level and allowed quantitative profiling and imaging of lymphocyte interactions (Dura et al., 2015). The device comprised an array of weir-based PDMS traps in a flow channel with each trap consisting of a front-side two-cell capture cup and back side single-cell capture cup. Furthermore, the device was treated with 7.5% bovine serum albumin or 10% pluronic F127. The device was utilized to assay the early molecular events during lymphocyte activation, to study the interaction between OT-I CD8 T cells and SIINFEKL-loaded MHCII-eGFP B cells and to characterize early activation dynamics of CD8 OT-1 T cells and TRP1 transnuclear T cells.

In a different attempt, Rosa et al., developed a microfluidic platform to study, in real-time, the dynamic interaction of flowing lymphocytes with adherent DCs (Moura Rosa et al., 2016). The platform, a PDMS based biochip placed on a glass substrate, had one main flow channel, two inlets and two outlets. The channel was coated with collagen or fibronectin to support cell adhesion. This device allowed the study of T cell-DC interactions and antigen-specific attachment of T cells to DCs and to modify in a controlled manner different parameters such as velocity, shear stress, deformation rates and migratory motility.

### Germinal Center Formation and Antibody Production

Currently, there are no experimental models that fully recapitulate the human humoral response in an *ex-vivo* lymph node microenvironment, probably due to the complexity of B cell differentiation and affinity maturation process in the germinal center reaction. The laboratory of Ankur Singh which has recently moved to Georgia Institute of Technology in the United States, has extensive work in the field; his group has generated mouse 3D synthetic immune organoids capable of regulating B cell development kinetics and of undergoing germinal center reactions that lead to the differentiation of antigen specific and high affinity germinal center B cells (Purwada et al., 2015, 2019; Purwada and Singh, 2017). Immune organoids are grown in 96-well plates and consist of mouse B primary cells embedded in a functionalized polymer; a Gelatin matrix activated with silicate nanoparticles or a four-armed poly (ethylene glycol) macromer that has maleimide groups at each terminus (PEG-4MAL) and that incorporates several adhesive peptides including Collagen-1 peptides. 3T3 (“3-days transfer, inoculum 3×10^5^ cells”) fibroblasts transduced with CD154, also called CD40 ligand (CD40L) and with B cell activating factor (BAFF) are included in the models to provide CD40L and BAFF signals which are secreted *in vivo* by stromal follicular dendritic cells (FDCs; (Purwada et al., 2019; Graney et al., 2020). Using their models, Singh et al. showed that B cells require “Enhancer of Zeste Homolog 2” (EZH2), a histone-lysine N-methyltransferase enzyme to form germinal centers and characterized the response against membrane bound versus soluble *Klebsiella pneumoniae* bacterial antigens (Béguelin et al., 2017; Graney et al., 2020). In addition, they demonstrated the role of integrin α4β1 and integrin αvβ3-binding ligands in inducing GL7+ (GC-like) and GL7- (non-GC-like) phenotype in differentiating B cells (Purwada et al., 2017). This attractive *ex-vivo* model could be improved by adding a microfluidic technology that would increase its potential applications.

Similarly, the laboratory of Donald Ingber at Harvard University, which has extensive work on Organs-on-chip, developed a PDMS-based microfluidic platform that supports the formation of lymph node follicles and germinal centers (Goyal et al., 2019). The platform consisted of two channels separated by a porous membrane whereby the lower channel hosted primary human B and T cells cultured within a gel composed of Matrigel and collagen type I and the upper channel served as a perfusion channel, continuously supplying cells with oxygen and nutrients. Ingber et,al showed that the formed lymphoid follicles and germinal centers support the differentiation of plasma cells when activated with IL-4 and CD40 agonistic antibody or when activated with bacteria (inactivated *S. aureus* Cowan I). In addition, they demonstrated that the introduction of a quadrivalent split virion influenza vaccine into the lymph node platform resulted in plasma cell formation, viral strain-specific anti-hemagglutinin immunoglobulin G (IgG) production, and cytokine secretion similar to that seen in serum of vaccinated humans.

### Lymph Node Malignancies

Some 3D models have focused on lymphoid malignancies with the aim of investigating the complex crosstalk between endothelial cells and immune tumor cells. Mannino et al reported a Lymphoma-on-a-chip model in which tumor cells were encapsulated in a hyaluronic acid-based hydrogel and traversed by a vascularized, perfusable, round microchannel (Mannino et al, 2017). This model would be useful for drug delivery studies as it allows the establishment of well-defined diffusion conditions and the evaluation of drug effects on endothelial cell permeability.

Shim et al. added a different degree of complexity to their developed microfluidic LN models by co-culturing lymph node tissue slices with tumor tissue slices in the same device to replicate communication between both organs on chip (Shim et al., 2019). Culture media circulated through both *ex-vivo* tissue samples and allowed the exchange of soluble factors. Authors found that the model replicated certain features of immune suppression mediated by the tumor, such as T cell activation (Shim et al., 2019).

There is extensive and growing literature describing cancer on chip models that recreate the tumor microenvironment and its interaction with the immune system, recently reviewed in (Maulana et al., 2021). These models represent a novel and attractive approach, closer to the *in vivo* situation than the classical ones, to address important issues in the field such as the tumor immune infiltrate, the immunosuppression mediated by the tumor microenvironment and the check point inhibitors, the drug resistance and the identification and evaluation of therapeutic drugs. Despite its relevance, we don’t describe these models here as it is out of the scope of the review, which is focused on lymph node on chips devices.

### Chemical Stimulation

To recreate stimulus-response behavior of the lymph node, Ross et al. developed a microfluidic device that hosts murine lymph node tissue slices and stimulated the different regions of the slices with a model therapeutic agent, namely, glucose-conjugated albumin (Ross et al., 2017). The microfluidic device consisted of a 3 layer PDMS in which the first layer constituted microchannels layer, the second layer constituted ports layer and the third layer constituted perfusion chamber. The results of the study demonstrated that the retention of the therapeutic agent was greater in the B-cell zone than it was in the T-cell zone.

## Lymph Node on Chip Applications

Despite the fact that most of the developed *in vitro* lymph node models have not been directly employed in pharmacological and toxicological applications, they do hold a huge potential to revolutionize drug discovery and drug development. In particular, the technology they are based upon, i.e. microfluidic technology, has enabled the recreation of intricate anatomical features of organs that closely resemble those *in vivo* in a manner that has not been possible otherwise. In addition, it allowed the design of “plug and play” components that are biologically relevant, simple and easy to use (Donoghue et al., 2021). On the other hand, 3D hydrogels with various extracellular matrix components, coupled with microfluidics, enabled more accurate representations of the cell-cell and cell-environment interactions compared to those in 2D culture plates. In addition, the ability to perfuse microfluidic chips allowed the shift from simple static systems into more dynamic ones in which cell medium and suspended biological material can be flowed, captured and recirculated. All these features make lymph nodes on-chip models, promising alternatives to traditional tissue culture plates. In addition they represent improved and attractive platforms for various applications in immunology, pathology, pharmacology and toxicology, as we will next describe. Although the review here focuses on models that mimic the lymph node physiology, there are other organ-on-chip models that are immune related and that hold promising potential for investigations related to the immune system (Stachowiak and Irvine, 2008; Jones et al., 2012; Dura et al., 2015; Sun et al., 2019; Fathi et al., 2020; Bachmann et al., 2021).

### Immunotoxicity Testing

Testing of potential adverse effects of human pharmaceuticals on immune cells is becoming a standard practice in drug development. As the initial accumulation of immune cells and drugs is often at the lymph node, *in vitro* systems mimicking both the anatomy and physiology of *in vivo* lymph node, will allow mechanistic studies that investigate drug efficacy, efficiency, and toxicity. Cell movement and viability can be easily tracked in real time as seen in some of the existing models described here and other parameters including immune cell proliferation, activation or the expression of soluble factors in the media can also be monitored. Alterations in these, along with antigen presentation, T cell activation, B cell differentiation and antibody production can be used as indicative factors of the immune cellular response to drugs. Fortunately, *in vitro* biomimetic lymph nodes-on-chip are very useful not only for evaluating the immunotoxicity of new immunotherapies, but also for addressing the toxicity generated by drugs already in the clinic.

### Immune Cellular Modulation

Lymph nodes-on-chip are of great benefit to medical fields that use drugs to target the immune system with the aim of modifying it and making it useful in the management of different disease conditions. In fact, immunotherapy has been used for long time in the treatment of allergy, to reduce the rejection of transplanted organs and to dampen autoimmunity (Boardman and Levings, 2019). Additionally, it has become a standard treatment for a variety of cancers. Cancer immunotherapy has been shown to induce durable responses in multiple solid and hematologic malignancies and the involvement of the draining lymph node is well documented (Fransen et al., 2018; van Pul et al., 2021). Moreover, it has been shown that immunotherapies also lead to unique toxicity profiles that are related to specific mechanisms of action (Kennedy and Salama, 2020). All these phenomena can be thoroughly investigated in physiologically relevant lymph nodes-on-chip platforms.

### Disease Modelling and Investigation

Lymph nodes-on-chip are attractive platforms for investigating diseases in which the immune system is involved such as cancer and inflammatory diseases. Normal and diseased cells or tissues can be loaded into biomimetic lymph node-on-chip platforms and their behavior can be easily monitored. Mechanisms of action of diseases as well as risk factors can be explored and novel pathways or elements can be identified. In addition, lymph nodes-on-chip can be connected to other organs-on-chip thus facilitating studies aimed at investigating the relationship between lymph nodes and other organs. For instance, people with inflammatory bowel disease have been shown to be at an increased risk of developing autoimmune diseases and inflammatory diseases (Halling et al., 2017). Such interesting links could be explored in multi-organ-on-chip platforms involving the lymph nodes which are known to host key immune cells. Such investigations provide a more systematic and holistic view of physiology, both in health and in disease.

### Vaccine Development

One of the major drawbacks associated with vaccine development is the lack of reliable and sensitive platforms to evaluate vaccine formulations in a physiologically relevant preclinical setting (Drake et al., 2012). Thus, lymph node-on-chip platforms are quite promising and advantageous systems for developing novel vaccines. Primary immune cells obtained from human donors can be loaded into the biomimetic *in vitro* lymph node systems along with novel vaccine formulations and their behavior and response accurately monitored. In specific, humoral immunity, antibody production and B cell-T cell interactions in response to vaccinations can be quantified.

### Personalized Medicine

While lymph node-on-chip models enable and accelerate drug and vaccine development processes, pending clinical acceptance, they also potentiate as platforms for personalized medicine. This is especially vital since genetic uniqueness might result in drug responses that differ across different populations. Patient immune cells can be obtained through blood sampling or minimally invasive liquid biopsy and be placed into developed lymph node-on-chip systems for evaluation. The possibility of including patient-derived immune cells in the device and replicating *ex-vivo* the cellular responses to different drugs opens new perspectives particularly in the precise and effective treatment of different diseases. For instance, resistance to cancer therapy remains one of the main challenges in the treatment of cancer (Mansoori et al., 2017). This phenomena can be addressed using lymph node-on-chip technology which allows for the direct incorporation of patient-derived cells in a physiologically relevant microenvironment and study of their specific cellular response to cancer therapy.

## Future Directions

Future lymph node on-chip designs will need to incorporate multiple design elements from foundational work into a complete, multifunctional lymph node. Microfluidic pathways, 3D cellular matrix, co-culture of multiple cell types, chemotaxis and cellular communication, all supported within a biologically sustainable microenvironment will make future lymph node devices more biologically relevant than ever before. Such lymph node models will allow investigations into different immune cellular events and their association with drug discovery and vaccine development. For example, they could be used for examining effects of pharmaceutical drugs on immune cell motility, antigen presentation, T cell activation, B cell differentiation and antibody production in a more realistic 3D scenario.

However, thorough validation of lymph nodes-on chip against both animal and clinical trial results is required to determine the reliability of these models as predictive tools and to define the extent to which these models represent human-relevant physiology (Hwang et al., 2021). Much remains to be done in this field and there are many opportunities to discover the possibilities lymph nodes on-chip can offer (Hwang et al., 2021). Yet, to ensure widespread adoption, it is necessary that the developed lymph node-on-chip platforms are easy to use, relatively inexpensive, and highly reproducible (Donoghue et al., 2021).

## References

[B1] Allen C. D. C. Okada T. Cyster J. G. (2007). Germinal-Center Organization and Cellular Dynamics. Immunity 27, 190–202. 10.1016/j.immuni.2007.07.009 17723214 PMC2242846

[B2] Bachmann B. Spitz S. Jordan C. Schuller P. Wanzenböck H. D. Haddadi B. (2021). Microvasculature-on-a-Chip: Bridging the Interstitial Blood-Lymph Interface via Mechanobiological Stimuli. Bioengineering. 10.1101/2021.04.08.438936

[B3] Béguelin W. Rivas M. A. Calvo Fernández M. T. Teater M. Purwada A. Redmond D. (2017). EZH2 Enables Germinal centre Formation through Epigenetic Silencing of CDKN1A and an Rb-E2f1 Feedback Loop. Nat. Commun. 8, 877. 10.1038/s41467-017-01029-x 29026085 PMC5638898

[B4] Birmingham K. G. O'Melia M. J. Bordy S. Reyes Aguilar D. El-Reyas B. Lesinski G. (2020). Lymph Node Subcapsular Sinus Microenvironment-On-A-Chip Modeling Shear Flow Relevant to Lymphatic Metastasis and Immune Cell Homing. iScience 23, 101751. 10.1016/j.isci.2020.101751 33241198 PMC7672279

[B5] Boardman D. A. Levings M. K. (2019). Cancer Immunotherapies Repurposed for Use in Autoimmunity. Nat. Biomed. Eng. 3, 259–263. 10.1038/s41551-019-0359-6 30952977

[B6] Byers A. M. Tapia T. M. Sassano E. R. Wittman V. (2009). *In Vitro* antibody Response to Tetanus in the MIMIC System Is a Representative Measure of Vaccine Immunogenicity. Biologicals 37, 148–151. 10.1016/j.biologicals.2009.02.018 19272794

[B7] Crivellato E. Vacca A. Ribatti D. (2004). Setting the Stage: an Anatomist's View of the Immune System. Trends Immunol. 25, 210–217. 10.1016/j.it.2004.02.008 15039048

[B8] Cupedo T. Stroock A. Coles M. (2012). Application of Tissue Engineering to the Immune System: Development of Artificial Lymph Nodes. Front. Immun. 3. 10.3389/fimmu.2012.00343 PMC3499788 23162557

[B9] Cyster J. G. (1999). Chemokines and Cell Migration in Secondary Lymphoid Organs. Science 286, 2098–2102. 10.1126/science.286.5447.2098 10617422

[B10] Dauner A. Agrawal P. Salvatico J. Tapia T. Dhir V. Shaik S. F. (2017). The *In Vitro* MIMIC Platform Reflects Age-Associated Changes in Immunological Responses after Influenza Vaccination. Vaccine 35, 5487–5494. 10.1016/j.vaccine.2017.03.099 28413134

[B11] Domeier P. P. Schell S. L. Rahman Z. S. M. (2017). Spontaneous Germinal Centers and Autoimmunity. Autoimmunity 50, 4–18. 10.1080/08916934.2017.1280671 28166685 PMC5669068

[B12] Donoghue L. Nguyen K. T. Graham C. Sethu P. (2021). Tissue Chips and Microphysiological Systems for Disease Modeling and Drug Testing. Micromachines 12, 139. 10.3390/mi12020139 33525451 PMC7911320

[B13] Drake D. R. Singh I. Nguyen M. N. Kachurin A. Wittman V. Parkhill R. (2012). In VitroBiomimetic Model of the Human Immune System for Predictive Vaccine Assessments. Disruptive Sci. Technol. 1, 28–40. 10.1089/dst.2012.0006

[B14] Dura B. Dougan S. K. Barisa M. Hoehl M. M. Lo C. T. Ploegh H. L. (2015). Profiling Lymphocyte Interactions at the Single-Cell Level by Microfluidic Cell Pairing. Nat. Commun. 6, 5940. 10.1038/ncomms6940 25585172

[B15] Fathi P. Holland G. Pan D. Esch M. B. (2020). Lymphatic Vessel on a Chip with Capability for Exposure to Cyclic Fluidic Flow. ACS Appl. Bio Mater. 3, 6697–6707. 10.1021/acsabm.0c00609 PMC13218302 35019335

[B16] Fransen M. F. Schoonderwoerd M. Knopf P. Camps M. G. M. Hawinkels L. J. A. C. Kneilling M. (2018). Tumor-draining Lymph Nodes Are Pivotal in PD-1/pd-L1 Checkpoint Therapy. JCI Insight 3, e124507. 10.1172/jci.insight.124507 PMC6328025 30518694

[B17] Fullerton J. N. Gilroy D. W. (2016). Resolution of Inflammation: a New Therapeutic Frontier. Nat. Rev. Drug Discov. 15, 551–567. 10.1038/nrd.2016.39 27020098

[B18] Galarza S. Kim H. Atay N. Peyton S. R. Munson J. M. (2020). 2D or 3D? How Cell Motility Measurements Are Conserved across Dimensions *In Vitro* and Translate *In Vivo* . Bioeng. Transl Med. 5. 10.1002/btm2.10148 PMC6971446 31989037

[B19] Gasteiger G. Ataide M. Kastenmüller W. (2016). Lymph Node - an Organ for T-Cell Activation and Pathogen Defense. Immunol. Rev. 271, 200–220. 10.1111/imr.12399 27088916

[B20] Giese C. Demmler C. D. Ammer R. Hartmann S. Lubitz A. Miller L. (2006). A Human Lymph Node *In Vitro*?Challenges and Progress. Artif. Organs 30, 803–808. 10.1111/j.1525-1594.2006.00303.x 17026580

[B21] Giese C. Lubitz A. Demmler C. D. Reuschel J. Bergner K. Marx U. (2010). Immunological Substance Testing on Human Lymphatic Micro-organoids *In Vitro* . J. Biotechnol. 148, 38–45. 10.1016/j.jbiotec.2010.03.001 20416346

[B22] Goyal G. Prabhala P. Mahajan G. Bausk B. Gilboa T. Xie L. (2019). Lymphoid Follicle Formation and Human Vaccination Responses Recapitulated in an Organ-On-A-Chip. Immunology. 10.1101/806505 PMC9109055 35289122

[B23] Graney P. L. Lai K. Post S. Brito I. Cyster J. Singh A. (2020). Organoid Polymer Functionality and Mode of *Klebsiella pneumoniae* Membrane Antigen Presentation Regulates *Ex Vivo* Germinal Center Epigenetics in Young and Aged B Cells. Adv. Funct. Mater. 30, 2001232. 10.1002/adfm.202001232 33692664 PMC7939142

[B24] Gretz J. E. Kaldjian E. P. Anderson A. O. Shaw S. (1996). Sophisticated Strategies for Information Encounter in the Lymph Node: the Reticular Network as a Conduit of Soluble Information and a Highway for Cell Traffic. J. Immunol. 157, 495–499. 8752893

[B25] Gretz J. E. Anderson A. O. Shaw S. (1997). Cords, Channels, Corridors and Conduits: Critical Architectural Elements Facilitating Cell Interactions in the Lymph Node Cortex. Immunol. Rev. 156, 11–24. 10.1111/j.1600-065X.1997.tb00955.x 9176696

[B26] Gretz J. E. Norbury C. C. Anderson A. O. Proudfoot A. E. I. Shaw S. (2000). Lymph-Borne Chemokines and Other Low Molecular Weight Molecules Reach High Endothelial Venules via Specialized Conduits while a Functional Barrier Limits Access to the Lymphocyte Microenvironments in Lymph Node Cortex. J. Exp. Med. 192, 1425–1440. 10.1084/jem.192.10.1425 11085745 PMC2193184

[B27] Haessler U. Kalinin Y. Swartz M. A. Wu M. (2009). An Agarose-Based Microfluidic Platform with a Gradient Buffer for 3D Chemotaxis Studies. Biomed. Microdevices 11, 827–835. 10.1007/s10544-009-9299-3 19343497

[B28] Haessler U. Pisano M. Wu M. Swartz M. A. (2011). Dendritic Cell Chemotaxis in 3D under Defined Chemokine Gradients Reveals Differential Response to Ligands CCL21 and CCL19. Proc. Natl. Acad. Sci. 108, 5614–5619. 10.1073/pnas.1014920108 21422278 PMC3078419

[B29] Hallfors N. Shanti A. Sapudom J. Teo J. Petroianu G. Lee S. (2021). Multi-Compartment Lymph-Node-On-A-Chip Enables Measurement of Immune Cell Motility in Response to Drugs. Bioengineering 8, 19. 10.3390/bioengineering8020019 33572571 PMC7912616

[B30] Halling M. L. Kjeldsen J. Knudsen T. Nielsen J. Hansen L. K. (2017). Patients with Inflammatory Bowel Disease Have Increased Risk of Autoimmune and Inflammatory Diseases. WJG 23, 6137–6146. 10.3748/wjg.v23.i33.6137 28970729 PMC5597505

[B31] Hassell B. A. Goyal G. Lee E. Sontheimer-Phelps A. Levy O. Chen C. S. (2017). Human Organ Chip Models Recapitulate Orthotopic Lung Cancer Growth, Therapeutic Responses, and Tumor Dormancy *In Vitro* . Cell Rep. 21, 508–516. 10.1016/j.celrep.2017.09.043 29020635

[B32] Higbee R. G. Byers A. M. Dhir V. Drake D. Fahlenkamp H. G. Gangur J. (2009). An Immunologic Model for Rapid Vaccine Assessment - A Clinical Trial in a Test Tube. Altern. Lab. Anim. 37, 19–27. 10.1177/026119290903701S05 19807200

[B33] Hingorani A. D. Kuan V. Finan C. Kruger F. A. Gaulton A. Chopade S. (2019). Improving the Odds of Drug Development success through Human Genomics: Modelling Study. Sci. Rep. 9, 18911. 10.1038/s41598-019-54849-w 31827124 PMC6906499

[B34] Hughes C. E. Benson R. A. Bedaj M. Maffia P. (2016). Antigen-Presenting Cells and Antigen Presentation in Tertiary Lymphoid Organs. Front. Immunol. 7. 10.3389/fimmu.2016.00481 PMC5097899 27872626

[B35] Hwang S.-H. Lee S. Park J. Y. Jeon J. S. Cho Y.-J. Kim S. (2021). Potential of Drug Efficacy Evaluation in Lung and Kidney Cancer Models Using Organ-On-A-Chip Technology. Micromachines 12, 215. 10.3390/mi12020215 33669950 PMC7924856

[B36] Jafarnejad M. Woodruff M. C. Zawieja D. C. Carroll M. C. Moore J. E. (2015). Modeling Lymph Flow and Fluid Exchange with Blood Vessels in Lymph Nodes. Lymphatic Res. Biol. 13, 234–247. 10.1089/lrb.2015.0028 PMC4685511 26683026

[B37] Jalili-Firoozinezhad S. Prantil-Baun R. Jiang A. Potla R. Mammoto T. Weaver J. C. (2018). Modeling Radiation Injury-Induced Cell Death and Countermeasure Drug Responses in a Human Gut-On-A-Chip. Cell Death Dis 9, 223. 10.1038/s41419-018-0304-8 29445080 PMC5833800

[B38] Jannat R. A. Robbins G. P. Ricart B. G. Dembo M. Hammer D. A. (2010). Neutrophil Adhesion and Chemotaxis Depend on Substrate Mechanics. J. Phys. Condens. Matter 22, 194117. 10.1088/0953-8984/22/19/194117 20473350 PMC2867619

[B39] Jones C. N. Dalli J. Dimisko L. Wong E. Serhan C. N. Irimia D. (2012). Microfluidic chambers for Monitoring Leukocyte Trafficking and Humanized Nano-Proresolving Medicines Interactions. Proc. Natl. Acad. Sci. 109, 20560–20565. 10.1073/pnas.1210269109 23185003 PMC3528552

[B40] Kaldjian E. P. Gretz J. E. Anderson A. O. Shi Y. Shaw S. (2001). Spatial and Molecular Organization of Lymph Node T Cell Cortex: a Labyrinthine Cavity Bounded by an Epithelium-like Monolayer of Fibroblastic Reticular Cells Anchored to Basement Membrane-like Extracellular Matrix. Int. Immunol. 13, 1243–1253. 10.1093/intimm/13.10.1243 11581169

[B41] Katakai T. (2004). A Novel Reticular Stromal Structure in Lymph Node Cortex: an Immuno-Platform for Interactions Among Dendritic Cells, T Cells and B Cells. Int. Immunol. 16, 1133–1142. 10.1093/intimm/dxh113 15237106

[B42] Kennedy L. B. Salama A. K. S. (2020). A Review of Cancer Immunotherapy Toxicity. CA A. Cancer J. Clin. 70, 86–104. 10.3322/caac.21596 31944278

[B43] Kraus T. Lubitz A. Schließer U. Giese C. Reuschel J. Brecht R. (2019). Evaluation of a 3D Human Artificial Lymph Node as Test Model for the Assessment of Immunogenicity of Protein Aggregates. J. Pharm. Sci. 108, 2358–2366. 10.1016/j.xphs.2019.02.011 30797781

[B44] Lian J. Luster A. D. (2015). Chemokine-guided Cell Positioning in the Lymph Node Orchestrates the Generation of Adaptive Immune Responses. Curr. Opin. Cell Biol. 36, 1–6. 10.1016/j.ceb.2015.05.003 26067148 PMC4639456

[B45] Liao S. von der Weid P. Y. (2015). Lymphatic System: an Active Pathway for Immune protection. Semin. Cell Develop. Biol. 38, 83–89. 10.1016/j.semcdb.2014.11.012 PMC4397130 25534659

[B46] Lin F. Butcher E. C. (2006). T Cell Chemotaxis in a Simple Microfluidic Device. Lab. Chip 6, 1462–1469. 10.1039/B607071J 17066171

[B47] Liu Y. Gill E. Shery Huang Y. Y. (2017). Microfluidic On-Chip Biomimicry for 3D Cell Culture: a Fit-For-Purpose Investigation from the End User Standpoint. Future Sci. OA 3, FSO173. 10.4155/fsoa-2016-0084 28670465 PMC5481809

[B48] Low L. A. Mummery C. Berridge B. R. Austin C. P. Tagle D. A. (2020). Organs-on-chips: into the Next Decade. Nat. Rev. Drug Discov. 20, 345–361. 10.1038/s41573-020-0079-3 32913334

[B49] Maharjan S. Cecen B. Zhang Y. S. (2020). 3D Immunocompetent Organ‐on‐a‐Chip Models. Small Methods 4, 2000235. 10.1002/smtd.202000235 33072861 PMC7567338

[B50] Mannino R. G. Santiago-Miranda A. N. Pradhan P. Qiu Y. Mejias J. C. Neelapu S. S. (2017). 3D Microvascular Model Recapitulates the Diffuse Large B-Cell Lymphoma Tumor Microenvironment *In Vitro* . Lab. Chip 17, 407–414. 10.1039/C6LC01204C 28054086 PMC5285444

[B51] Mansoori B. Mohammadi A. Davudian S. Shirjang S. Baradaran B. (2017). The Different Mechanisms of Cancer Drug Resistance: A Brief Review. Adv. Pharm. Bull. 7, 339–348. 10.15171/apb.2017.041 29071215 PMC5651054

[B52] Martino M. M. Hubbell J. A. (2010). The 12th-14th Type III Repeats of Fibronectin Function as a Highly Promiscuous Growth Factor-Binding Domain. FASEB J. 24, 4711–4721. 10.1096/fj.09-151282 20671107

[B53] Maulana T. I. Kromidas E. Wallstabe L. Cipriano M. Alb M. Zaupa C. (2021). Immunocompetent Cancer-On-Chip Models to Assess Immuno-Oncology Therapy. Adv. Drug Deliv. Rev. 173, 281–305. 10.1016/j.addr.2021.03.015 33798643

[B54] Mcknight A. J. Gordon S. (1998). “Membrane Molecules as Differentiation Antigens of Murine Macrophages,” in Advances in Immunology (Elsevier), 271–314. 10.1016/S0065-2776(08)60562-3 9505092

[B55] Mitra B. Jindal R. Lee S. Dong D. X. Li L. Sharma N. (2013). Microdevice Integrating Innate and Adaptive Immune Responses Associated with Antigen Presentation by Dendritic Cells. RSC Adv. 3, 16002–16010. 10.1039/C3RA41308J 29682279 PMC5909707

[B56] Moura Rosa P. Gopalakrishnan N. Ibrahim H. Haug M. Halaas Ø. (2016). The Intercell Dynamics of T Cells and Dendritic Cells in a Lymph Node-On-A-Chip Flow Device. Lab. Chip 16, 3728–3740. 10.1039/C6LC00702C 27560793

[B57] Nam K.-H. Smith A. S. T. Lone S. Kwon S. Kim D.-H. (2015). Biomimetic 3D Tissue Models for Advanced High-Throughput Drug Screening. J. Lab. Autom. 20, 201–215. 10.1177/2211068214557813 25385716 PMC4459652

[B58] Nandagopal S. Wu D. Lin F. (2011). Combinatorial Guidance by CCR7 Ligands for T Lymphocytes Migration in Co-existing Chemokine Fields. PLoS ONE 6, e18183. 10.1371/journal.pone.0018183 21464944 PMC3064588

[B59] Novkovic M. Onder L. Bocharov G. Ludewig B. (2020). Topological Structure and Robustness of the Lymph Node Conduit System. Cell Rep. 30, 893–904. 10.1016/j.celrep.2019.12.070 31968261

[B60] Pape K. A. Catron D. M. Itano A. A. Jenkins M. K. (2007). The Humoral Immune Response Is Initiated in Lymph Nodes by B Cells that Acquire Soluble Antigen Directly in the Follicles. Immunity 26, 491–502. 10.1016/j.immuni.2007.02.011 17379546

[B61] Parlato S. De Ninno A. Molfetta R. Toschi E. Salerno D. Mencattini A. (2017). 3D Microfluidic Model for Evaluating Immunotherapy Efficacy by Tracking Dendritic Cell Behaviour toward Tumor Cells. Sci. Rep. 7, 1093. 10.1038/s41598-017-01013-x 28439087 PMC5430848

[B62] Pelletier A. J. van der Laan L. J. W. Hildbrand P. Siani M. A. Thompson D. A. Dawson P. E. (2000). Presentation of Chemokine SDF-1α by Fibronectin Mediates Directed Migration of T Cells. Blood 96, 2682–2690. 10.1182/blood.v96.8.2682.h8002682_2682_2690 11023498

[B63] Polini A. Del Mercato L. L. Barra A. Zhang Y. S. Calabi F. Gigli G. (2019). Towards the Development of Human Immune-System-On-A-Chip Platforms. Drug Discov. Today 24, 517–525. 10.1016/j.drudis.2018.10.003 30312743 PMC6440212

[B64] Purwada A. Jaiswal M. K. Ahn H. Nojima T. Kitamura D. Gaharwar A. K. (2015). *Ex Vivo* engineered Immune Organoids for Controlled Germinal center Reactions. Biomaterials 63, 24–34. 10.1016/j.biomaterials.2015.06.002 26072995 PMC4490011

[B65] Purwada A. Shah S. B. Béguelin W. August A. Melnick A. M. Singh A. (2019). *Ex Vivo* synthetic Immune Tissues with T Cell Signals for Differentiating Antigen-specific, High Affinity Germinal center B Cells. Biomaterials 198, 27–36. 10.1016/j.biomaterials.2018.06.034 30041943 PMC6355359

[B66] Purwada A. Shah S. B. Beguelin W. Melnick A. M. Singh A. (2017). Modular Immune Organoids with Integrin Ligand Specificity Differentially Regulate *Ex Vivo* B Cell Activation. ACS Biomater. Sci. Eng. 3, 214–225. 10.1021/acsbiomaterials.6b00474 33450794

[B67] Purwada A. Singh A. (2017). Immuno-engineered Organoids for Regulating the Kinetics of B-Cell Development and Antibody Production. Nat. Protoc. 12, 168–182. 10.1038/nprot.2016.157 28005068 PMC6355337

[B68] Qian F. Huang C. Lin Y.-D. Ivanovskaya A. N. O'Hara T. J. Booth R. H. (2017). Simultaneous Electrical Recording of Cardiac Electrophysiology and Contraction on Chip. Lab. Chip 17, 1732–1739. 10.1039/C7LC00210F 28448074

[B69] Radke L. Sandig G. Lubitz A. Schließer U. von Horsten H. Blanchard V. (2017). *In Vitro* Evaluation of Glycoengineered RSV-F in the Human Artificial Lymph Node Reactor. Bioengineering 4, 70. 10.3390/bioengineering4030070 PMC5615316 28952549

[B70] Rana M. K. Srivastava J. Yang M. Chen C. S. Barber D. L. (2015). Hypoxia Increases Extracellular Fibronectin Abundance but Not Assembly during Epithelial Cell Transdifferentiation. J. Cell Sci. 128, 1083–1089. 10.1242/jcs.155036 25616899 PMC4359919

[B71] Ricart B. G. John B. Lee D. Hunter C. A. Hammer D. A. (2011). Dendritic Cells Distinguish Individual Chemokine Signals through CCR7 and CXCR4. J.I. 186, 53–61. 10.4049/jimmunol.1002358 21106854

[B72] Richardson L. Kim S. Menon R. Han A. (2020). Organ-On-Chip Technology: The Future of Feto-Maternal Interface Research? Front. Physiol. 11, 715. 10.3389/fphys.2020.00715 32695021 PMC7338764

[B73] Roberts R. A. Kavanagh S. L. Mellor H. R. Pollard C. E. Robinson S. Platz S. J. (2014). Reducing Attrition in Drug Development: Smart Loading Preclinical Safety Assessment. Drug Discov. Today 19, 341–347. 10.1016/j.drudis.2013.11.014 24269835

[B74] Ross A. E. Belanger M. C. Woodroof J. F. Pompano R. R. (2017). Spatially Resolved Microfluidic Stimulation of Lymphoid Tissue *Ex Vivo* . Analyst 142, 649–659. 10.1039/C6AN02042A 27900374 PMC7863610

[B75] Ross A. E. Pompano R. R. (2018). Diffusion of Cytokines in Live Lymph Node Tissue Using Microfluidic Integrated Optical Imaging. Analytica Chim. Acta 1000, 205–213. 10.1016/j.aca.2017.11.048 29289312

[B76] Sainte-Marie G. (2010). The Lymph Node Revisited: Development, Morphology, Functioning, and Role in Triggering Primary Immune Responses. Anat. Rec. 293, 320–337. 10.1002/ar.21051 20101739

[B77] Sardi M. Lubitz A. Giese C. (2016). Modeling Human Immunity *In Vitro*: Improving Artificial Lymph Node Physiology by Stromal Cells. Appl. Vitro Toxicol. 2, 143–150. 10.1089/aivt.2016.0004

[B78] Schudel A. Francis D. M. Thomas S. N. (2019). Material Design for Lymph Node Drug Delivery. Nat. Rev. Mater. 4, 415–428. 10.1038/s41578-019-0110-7 32523780 PMC7286627

[B79] Shanti A. Samara B. Abdullah A. Hallfors N. Accoto D. Sapudom J. (2020). Multi-Compartment 3D-Cultured Organ-On-A-Chip: Towards a Biomimetic Lymph Node for Drug Development. Pharmaceutics 12, 464. 10.3390/pharmaceutics12050464 PMC7284904 32438634

[B80] Shanti A. Teo J. Stefanini C. (2018). *In Vitro* Immune Organs-On-Chip for Drug Development: A Review. Pharmaceutics 10, 278. 10.3390/pharmaceutics10040278 PMC6320867 30558264

[B81] Shim K.-Y. Lee D. Han J. Nguyen N.-T. Park S. Sung J. H. (2017). Microfluidic Gut-On-A-Chip with Three-Dimensional Villi Structure. Biomed. Microdevices 19, 37. 10.1007/s10544-017-0179-y 28451924

[B82] Shim S. Belanger M. C. Harris A. R. Munson J. M. Pompano R. R. (2019). Two-way Communication Betweenex Vivotissues on a Microfluidic Chip: Application to Tumor-Lymph Node Interaction. Lab. Chip 19, 1013–1026. 10.1039/C8LC00957K 30742147 PMC6416076

[B83] Sonmez U. M. Wood A. Justus K. Jiang W. Syed-Picard F. LeDuc P. R. (2020). Chemotactic Responses of Jurkat Cells in Microfluidic Flow-free Gradient Chambers. Micromachines 11, 384. 10.3390/mi11040384 PMC7231302 32260431

[B84] Stachowiak A. N. Irvine D. J. (2008). Inverse Opal Hydrogel-Collagen Composite Scaffolds as a Supportive Microenvironment for Immune Cell Migration. J. Biomed. Mater. Res. 85A, 815–828. 10.1002/jbm.a.31661 17937415

[B85] Stucki J. D. Hobi N. Galimov A. Stucki A. O. Schneider-Daum N. Lehr C.-M. (2018). Medium Throughput Breathing Human Primary Cell Alveolus-On-Chip Model. Sci. Rep. 8, 14359. 10.1038/s41598-018-32523-x 30254327 PMC6156575

[B86] Sun W. Luo Z. Lee J. Kim H.-J. Lee K. Tebon P. (2019). Organ-on-a-Chip for Cancer and Immune Organs Modeling. Adv. Healthc. Mater. 8, 1801363. 10.1002/adhm.201801363 PMC6424124 30605261

[B87] van Pul K. M. Fransen M. F. van de Ven R. de Gruijl T. D. (2021). Immunotherapy Goes Local: The Central Role of Lymph Nodes in Driving Tumor Infiltration and Efficacy. Front. Immunol. 12, 643291. 10.3389/fimmu.2021.643291 33732264 PMC7956978

[B88] Vernetti L. A. Senutovitch N. Boltz R. DeBiasio R. Ying Shun T. Gough A. (2016). A Human Liver Microphysiology Platform for Investigating Physiology, Drug Safety, and Disease Models. Exp. Biol. Med. (Maywood) 241, 101–114. 10.1177/1535370215592121 26202373 PMC4723301

[B89] von Andrian U. H. Mempel T. R. (2003). Homing and Cellular Traffic in Lymph Nodes. Nat. Rev. Immunol. 3, 867–878. 10.1038/nri1222 14668803

[B90] Warren W. Drake D. Moser J. Singh I. Song H. Mishkin E. (2007). Co-culture Lymphoid Tissue Equivalent (Lte) for an Artificial Immune System (Ais).

[B91] Warren W. Fahlenkamp H. Russell H. Kachurin A. Li C. Nguyen M. (2005). Artificial Immune System: Methods for Making and Use.

[B92] Wiig H. Keskin D. Kalluri R. (2010). Interaction between the Extracellular Matrix and Lymphatics: Consequences for Lymphangiogenesis and Lymphatic Function. Matrix Biol. 29, 645–656. 10.1016/j.matbio.2010.08.001 20727409 PMC3992865

[B93] Woods A. Longley R. L. Tumova S. Couchman J. R. (2000). Syndecan-4 Binding to the High Affinity Heparin-Binding Domain of Fibronectin Drives Focal Adhesion Formation in Fibroblasts. Arch. Biochem. Biophys. 374, 66–72. 10.1006/abbi.1999.1607 10640397

[B94] Zaretsky I. Polonsky M. Shifrut E. Waysbort N. Friedman N. Aidelberg G. (2012). Monitoring the Dynamics of Primary T Cell Activation and Differentiation Using Long Term Live Cell Imaging in Microwell Arrays. Lab. Chip 12, 5007. 10.1039/c2lc40808b 23072772

